# Graphene Oxide Quantum Dots Reduce Oxidative Stress and Inhibit Neurotoxicity In Vitro and In Vivo through Catalase‐Like Activity and Metabolic Regulation

**DOI:** 10.1002/advs.201700595

**Published:** 2018-03-04

**Authors:** Chaoxiu Ren, Xiangang Hu, Qixing Zhou

**Affiliations:** ^1^ Key Laboratory of Pollution Processes and Environmental Criteria (Ministry of Education) Tianjin Key Laboratory of Environmental Remediation and Pollution Control College of Environmental Science and Engineering Nankai University Tianjin 300071 China

**Keywords:** catalase‐like activity, graphene oxide, metabolomics, neuroprotection, quantum dots

## Abstract

Both oxidative stress and neurotoxicity are huge challenges to human health, and effective methods and agents for resisting these adverse effects are limited, especially in vivo. It is shown here that, compared to large graphene oxide (GO) nanosheets, GO quantum dots (GOQDs), as nanozymes, efficiently reduce reactive oxygen species (ROS) and H_2_O_2_ in 1‐methyl‐4‐phenyl‐pyridinium ion (MPP^+^)‐induced PC12 cells. In addition, GOQDs exert neuroprotective effects in a neuronal cell model by decreasing apoptosis and α‐synuclein. GOQDs also efficiently diminish ROS, apoptosis, and mitochondrial damage in zebrafish treated with MPP^+^. Furthermore, GOQDs‐pretreated zebrafish shows increased locomotive activity and Nissl bodies in the brain, confirming that GOQDs ameliorate MPP^+^‐induced neurotoxicity, in contrast to GO nanosheets. GOQDs contribute to neurotoxic amelioration by increasing amino acid metabolism, decreasing tricarboxylic acid cycle activity, and reducing steroid biosynthesis, fatty acid biosynthesis, and galactose metabolic pathway activity, which are related to antioxidation and neurotransmission. Meanwhile, H_2_O_2_ decomposition and Fenton reactions suggest the catalase‐like activity of GOQDs. GOQDs can translocate into zebrafish brains and exert catalase‐mimicking activity to resist oxidation in the intracellular environment. Unlike general nanomaterials, biocompatible GOQDs demonstrate their high potential for human health by reducing oxidative stress and inhibiting neurotoxicity.

## 1 Introduction

Neurotoxicity has severe influences on human health and has pathological features, such as memory disorders, learning ability decline, behavioral dysfunction, and cognitive dysfunction.^1^ Although several therapeutic agents, such as epigallocatechin‐3‐gallate, anthocyanins, sialic acid, and thioflavin, have been proposed to mitigate neurotoxicity, poor chemical stability, loss of activation in harsh environments, and substandard bioavailability limit the use of these agents.^2^ Nanomaterials have several unique physicochemical characteristics for overcoming the above drawbacks: a small size for crossing the blood–brain barrier (BBB); an ultrahigh surface‐area‐to‐volume ratio and specific surface chemistry for enhancing biocompatibility and binding affinity; an increased resistance to biodegradation; a decreased susceptibility to denaturation; and an ability to mimic the cellular matrix in natural tissues or body fluid, in a manner that is similar to enzymes and proteins, for therapeutic applications.^3^

Quantum dots (QDs) are widely used in nanomedicine due to their narrow and symmetric emission spectrum, high water‐solubility, and high bioavailability.^4^ In addition, multiple reports have shown that graphene oxide (GO) can cross the BBB into the brain and that GO can be internalized by the cell to ultimately translocate into the nucleus.^5^ Although GO itself triggers neurotoxicity by translocating from water to the zebrafish brain,[[qv: 5b]] modified GO has attracted considerable attention in the clinic due to the enzyme‐like activities and biocompatibility of modified GO.^6^ GO quantum dots (GOQDs), which are smaller versions of GO, combine the virtues of GO and QDs and have fluorescent characteristics, biocompatibility, low cytotoxicity, and antioxidant nanozyme‐like activity.^7^ Here, GOQDs were employed to test their advantages against neurotoxicity in vitro and in vivo, as this has not been previously investigated.

The compound 1‐methyl‐4‐phenyl‐pyridinium ion (MPP^+^) induces oxidative stress and apoptosis in PC12 cells, which were previously used as a model of neurotoxicity in vitro.^8^ The zebrafish is an excellent model for studying neurotoxicity because the nervous system shares many similarities with the human nervous system.^9^ Thus, PC12 cells and larval zebrafish were chosen to investigate the protective roles of GOQDs on MPP^+^‐induced neurotoxicity in vitro and in vivo, respectively. The specific molecular mechanisms were also elucidated. This study provides new insights into the treatment of neurological disorders using nanomaterials.

## 2 Results

### 2.1 Cell Viability and Morphology

**Figure 1:**
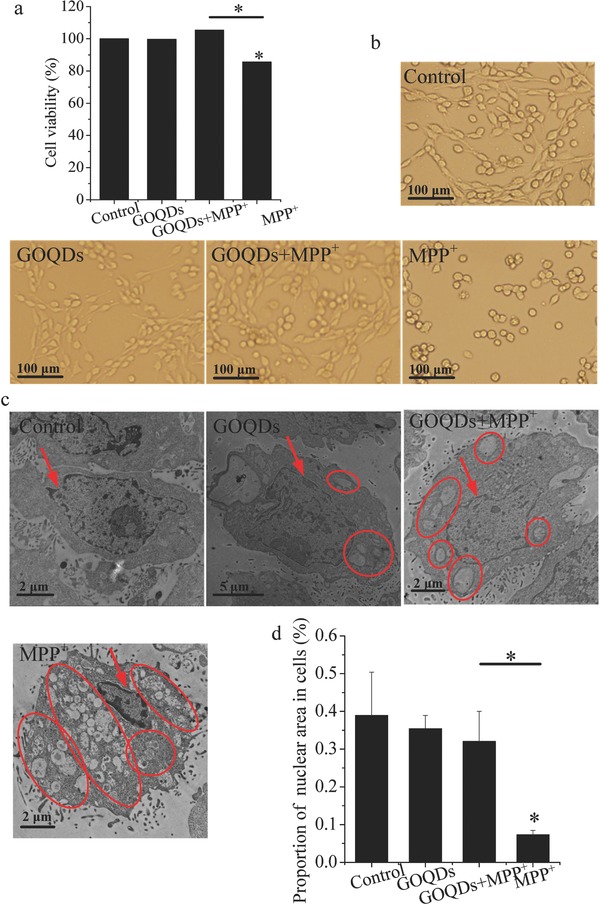
Cell viability and morphology. a) Cell viability of PC12 cells. b) Cell morphology of PC12 cells. c) TEM images of nuclear morphology and vesicles in PC12 cells. Red arrows indicate nuclei. Red circles denote vesicles. d) Ratios of nuclear area to cell area. *P < 0.05, compared with the control. * *P* < 0.05, GOQDs‐pretreated group compared with the MPP^+^‐treated group.

The characteristics of GOQDs and the optimization of the test concentrations of GOQDs (100 µg mL^−1^) and MPP^+^ (4 × 10^−3^ m) for cells are presented in the Supporting Information (Figures S1 and S2, Supporting Information). Cell viability significantly increased by 20% in GOQDs‐pretreated cells compared with MPP^+^‐treated cells, as shown in **Figure** **1**a. As shown in Figure 1b, normal PC12 cells exhibited a polygon shape, and their edges were intact and clear. The cells were shrunken and displayed a round shape after MPP^+^ exposure. However, GOQDs‐pretreated PC12 cells exhibited normal morphology. The cell nuclei in the control group were plump and homogeneous, as denoted by red arrows, and no vesicles were observed (Figure 1c). The cell nuclei showed crenation and condensation, and multiple vesicles were generated in the MPP^+^‐exposed cells; the nuclei and vesicles are denoted by red arrows and red circles, respectively, in Figure 1c. However, the nuclear area was 357% larger in the GOQDs‐pretreated cells than in the MPP^+^‐treated cells, and vesicle production was reduced in the GOQDs‐pretreated cells relative to the MPP^+^‐treated cells (Figure 1c,d).

### 2.2 Neuropathological Responses In Vitro

**Figure 2:**
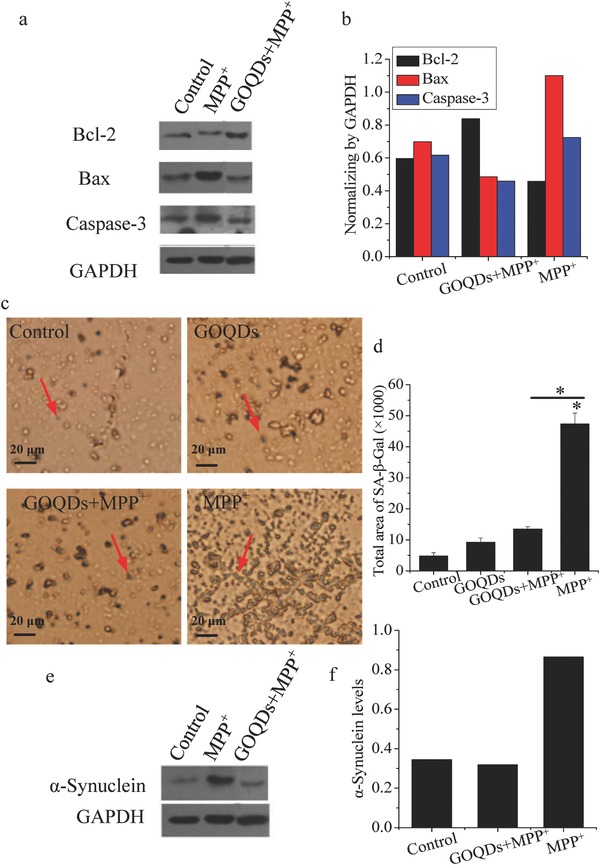
Effects of GOQDs on MPP^+^‐induced neuropathological responses in vitro. a) Western blotting bands of Bcl‐2, Bax, and caspase‐3. b) Bcl‐2, Bax, and caspase‐3 were normalized to glyceraldehyde‐3‐phosphate dehydrogenase (GAPDH) according to the intensity of the Western blotting bands. c) Senescent cells stained with senescence‐associated β‐galactosidase. The blue cells indicated by red arrows are senescent cells. d) Quantification of senescent cells. e) Western blotting bands of α‐synuclein. f) α‐synuclein was normalized to GAPDH according to the intensity of Western blotting bands. *P < 0.05, compared with the control. * *P* < 0.05, GOQDs‐pretreated group compared with the MPP^+^‐treated group.

Compared with the control cells, MPP^+^ cells showed a 275% increase in reactive oxygen species (ROS) levels, whereas no significant differences were evident between the GOQDs‐pretreated and control groups (Figure S3a,b, Supporting Information). Compared with the controls, the hydrogen peroxide (H_2_O_2_) levels increased by 55.6% in the MPP^+^ group but were significantly decreased by 35.7% in the PC12 cells pretreated with GOQDs compared with MPP^+^ alone (Figure S3c, Supporting Information). No significant difference was evident in the H_2_O_2_ levels between the GOQDs‐pretreated and control groups. Because a ROS increase could promote apoptosis,^10^ apoptosis biomarkers (Bcl‐2, Bax and caspase‐3) were analyzed. Compared with the controls, MPP^+^ exposure resulted in increases in Bax and caspase‐3 and a decrease in Bcl‐2 (**Figure** **2**a,b). The above results were consistent with a previous report, though no statistical analysis was performed.^11^ The above alterations were mitigated by pretreatment with GOQDs. Compared with the controls, the areas of senescence indicated by β‐galactosidase staining (SA‐β‐Gal) increased by 891% in the MPP^+^ group (Figure 2c,d). When the GOQDs pretreatment was performed before the MPP^+^ exposure, the senescent appearance was ameliorated, and the areas of SA‐β‐Gal‐positive cells were significantly decreased by 72% (Figure 2c,d). α‐Synuclein is a major component of Lewy bodies, which are associated with functional neuronal decline and neurodegenerative disease.^12^ Compared to the control, α‐Synuclein increased after administration of MPP^+^ (Figure 2e,f), which was consist with the results of the study by Zhang et al. and supported the reliability of the Western blotting analysis without the statistical analysis.[[qv: 3c]] However, α‐synuclein in the MPP^+^‐treated cells decreased due to the GOQDs pretreatment.

### 2.3 Metabolomics Analysis In Vitro

**Figure 3:**
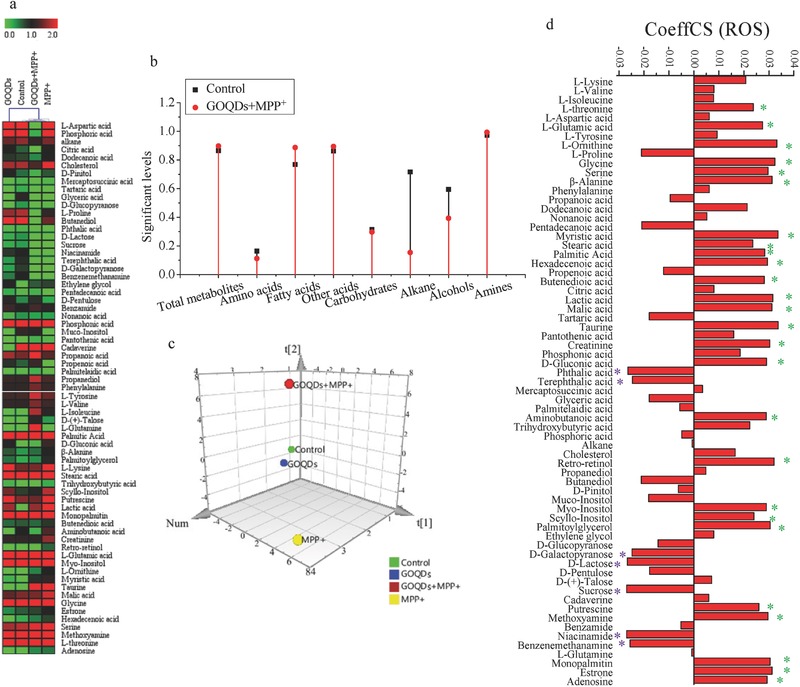
Effects of GOQDs on metabolomics in vitro induced by MPP^+^. a) Heat maps of identified metabolites. b) Significant levels of metabolites in the control and GOQDs+MPP^+^ groups compared with the MPP^+^ group. c) Metabolic cluster analysis using a PCA scores plot. d) CoeffCS of metabolites as the *X* variable and ROS as the *Y* variable by PLS analysis. The metabolites labeled by asterisks represent the metabolites with a VIP greater than one. The metabolites labeled with green and purple asterisks represent the metabolites that positively and negatively contribute to ROS, respectively. PCA, principal component analysis; PLS, partial least squares; CoeffCS, coefficient centered and scaled X data (metabolites); VIP, variable importance for the projection; ROS, reactive oxide species.

The relative abundances of the metabolites are presented using heat maps (**Figure** **3**a). The metabolic profiles were divided into two groups by hierarchical clustering (HCL) analysis, namely, the control/GOQDs/GOQDs+MPP^+^ and MPP^+^ groups, demonstrating that MPP^+^ affected the metabolic profiles; however, these alterations were mitigated by the GOQDs pretreatment. The differences among all tested groups (Figure 3b) and the principal component analysis (PCA) score plots (Figure 3c) also confirmed that the metabolic disturbances in the MPP^+^‐induced cells were mitigated by the GOQD pretreatment. Moreover, the associations between metabolism and ROS were analyzed using the partial least squares (PLS) model with ROS as the *Y* variable and the metabolic levels as the *X* variables (Figure 3d). The metabolites with variable importance for the projection (VIP) values greater than 1 are labeled by asterisks (Figure 3d). The metabolites labeled with green and purple asterisks represent the metabolites that had significant positive and negative contributions to ROS, respectively. In addition, the correlations between the changes in pathophysiological indicators (cell viability, H_2_O_2_ levels, α‐synuclein, Bcl‐2, Bax, and caspase‐3) and the corresponding two metabolites with the largest VIP values were analyzed by linear fitting in vitro. *R* ^2^ was from 0.77 to 0.99, suggesting the close correlation between pathophysiological changes and metabolite regulation (Figure S4, Supporting Information). For example, α‐synuclein was positively correlated with L‐lysine (*R* ^2^ = 0.99) and trihydroxybutyric acid (*R* ^2^ = 0.99), and this correlation was supported by studies indicating that the interaction with L‐lysine accelerated the rate of α‐synuclein fibrillation.^13^ In this study, α‐synuclein, L‐lysine, and trihydroxybutyric acid were all downregulated by the GOQDs pretreatment compared with the MPP^+^‐treated group, indicating that the regulation of metabolites by GOQDs was an important approach to mitigating neurotoxicity.

### 2.4 Catalase‐Like Activity of the GOQDs

Gas bubbles were observed after H_2_O_2_ was incubated with catalase or GOQDs (Figure S5a, Supporting Information), demonstrating that the GOQDs had similar functions to those of catalase to catalytically decompose H_2_O_2_ to H_2_O and O_2_.^14^ Furthermore, the enzymatic activities of GOQDs and catalase were quantified. After 25 mmol L^−1^ H_2_O_2_ was decomposed by the GOQDs and catalase, the final H_2_O_2_ concentrations were 2.7 and 2.2 mmol L^−1^, respectively (Figure S5b, Supporting Information). This result indicated that the enzymatic activity of 100 µg mL^−1^ GOQDs was almost comparable to that of 4 U mL^−1^ catalase. Moreover, the catalase‐like activity of the GOQDs was confirmed with Fenton reactions. Hydroxyl radicals (•OH) were significantly inhibited by the GOQDs and catalase (Figure S5c, Supporting Information).

### 2.5 Uptake of Nanomaterials In Vivo

**Figure 4:**
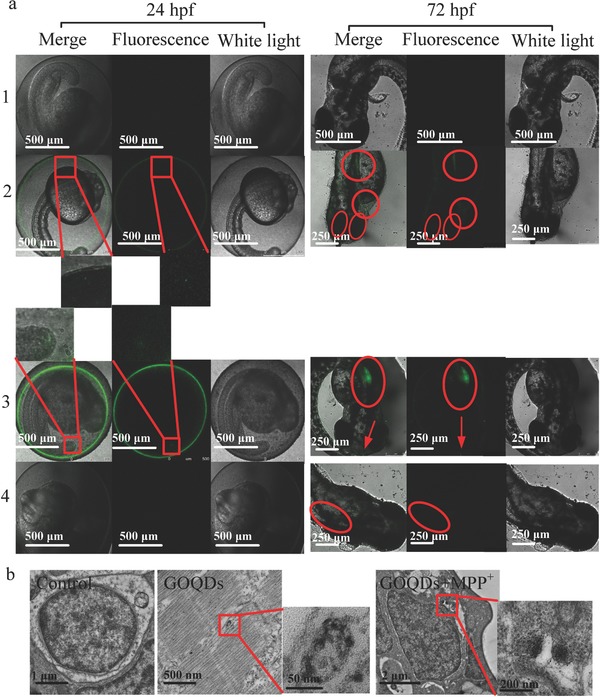
Translocation of GOQDs to zebrafish. a) LSCM images of zebrafish treated with E3 medium (1), FITC‐GOQDs (2), FITC (3), and GOQDs (4) at 24 and 72 hpf. The green fluorescence dots in the embryos at 24 hpf are denoted by red rectangles, and the enlarged ones are shown below. The green fluorescence in the larvae at 72 hpf is denoted by red arrows and circles. b) TEM images of cell brains in zebrafish. Red rectangles indicate the black dots (GOQDs) in the cells, and the pictures on the right are enlarged.

To verify whether the GOQDs could translocate from the water environment into zebrafish, we used fluorescein isothiocyanate (FITC) immobilized on GOQDs to trace the GOQDs by green fluorescence. The absorption curve and peak locations of the FITC‐GOQDs were comparable to those of FITC, which indicated that the GOQDs were successfully labeled with FITC (Figure S6, Supporting Information). At 24 h postfertilization (hpf), green fluorescence was found on the surface of the chorion (green circle) and in the yolk sac edema (green dots denoted by the red box) in the embryo treated with the FITC‐GOQDs (**Figure** **4**a). In addition, green fluorescence was found on the surface of the chorion (green circle) and in the tail (green dots denoted by the red box) in the embryo treated with FITC (Figure 4a). The results indicated that the GOQDs and FITC localized to different regions within the embryo and that the GOQDs could not only adhere to the surface of the chorion but also translocate from the water to the embryo and concentrate in the yolk sac edema. A previous report have shown that GO adheres to the chorion of the zebrafish embryo mainly via hydroxyl group interactions and crosses the chorion of the zebrafish through independent pore canals via passive diffusion.^15^ At 72 hpf, the green fluorescence denoted by the red circles was distributed to the head, eyes, gills, heart, intestines, and yolk sac edema of the larvae treated with the FITC‐GOQDs, suggesting that the GOQDs were predominantly distributed to these regions (Figure 4a), especially in the heart and intestines because the blood circulatory system would most likely be the first organ to be exposed to carbon‐based nanoparticles^16^ and because swallowing into the intestines is also a common pathway for absorbing nanoparticles.[[qv: 5b]] However, the green fluorescence denoted by the red circles was only distributed in the yolk sac edema of the larvae treated with FITC, indicating that the GOQDs and FITC concentrated in different regions within the larvae (Figure 4a). Overall, the GOQDs translocated from the water to the zebrafish regardless of whether the chorion was present. At 72 hpf, the larval zebrafish incubated with the GOQDs also displayed dim green fluorescence, which may be attributed to the natural fluorescence of the GOQDs. Compared with the controls, black dots in transmission electron microscopy (TEM) sections were observed in the cytoplasm of the brain cells in the GOQDs‐treated and pretreated zebrafish (Figure 4b). Our previous report confirmed that GO nanosheets translocated to the brains of zebrafish,[[qv: 5b]] and a previous study proposed that GO was able to cross biological barriers through an energy‐independent passive mechanism.^17^ Meanwhile, a previous study reported that the cellular internalization levels of GOQDs were ≈10–80‐fold higher than those of larger GO nanosheets.^18^ These results confirmed that the GOQDs translocated to the brains of the zebrafish, which was essential for GOQDs‐mediated protection of the zebrafish from MPP^+^‐induced neurotoxicity.

### 2.6 Developmental Toxicity and Locomotive Activity In Vivo

**Figure 5:**
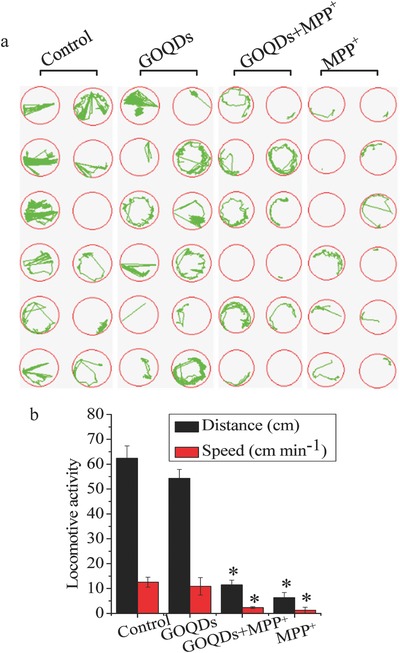
Effects of GOQDs on behavioral disturbances of zebrafish induced by MPP^+^. a) Spontaneous movement trajectories of larvae in 96‐well plates treated with MPP^+^ with or without preincubation with GOQDs. The green curves represent the movement trajectories of the larvae. b) Distances and speeds of the larvae. *P < 0.05, compared with the control.

Compared with the small (diameter, 2–5 nm) GOQDs,^19^ the GOQDs used in this study (lateral sizes, 20–40 nm; thicknesses, 4.18–5.19 nm) at 100 µg mL^−1^ were biocompatible (Figure S2c, Supporting Information), demonstrating that size determined nanomaterial biocompatibility; the two QDs had different sizes, which was a key factor that affected their toxicities. Therefore, in this study, the optimization of the test concentrations of the GOQDs (100 µg mL^−1^) and MPP^+^ (1.5 × 10^−3^ m) for zebrafish was performed as is presented in the Supporting Information (Figure S2c,d, Supporting Information). Several developmental malformations, including spinal curvature, rumplessness, and pericardial/yolk sac edema, were caused by MPP^+^, as shown in Figure S7a (Supporting Information). Although both the GOQDs‐pretreated and MPP^+^‐treated groups showed high mortality and malformation rates compared to the control group, the malformation rates induced by MPP^+^ were reduced by 24.8% with the pretreatment of GOQDs (Figure S7b, Supporting Information). The neurological functions in developmental neurotoxicity were determined with a commonly used approach, namely, evaluation of locomotion. The results showed that the speeds of the control and MPP^+^‐treated zebrafish were 12.5, 1.3, and 2.3 cm min^−1^. The swimming speeds increased by 77% after GOQDs pretreatment compared to the MPP^+^‐treated zebrafish, suggesting that the pretreatment with GOQDs mitigated the damage to locomotive activity induced by MPP^+^ (**Figure** **5**a,b).

### 2.7 Neuropathological Responses In Vivo

**Figure 6:**
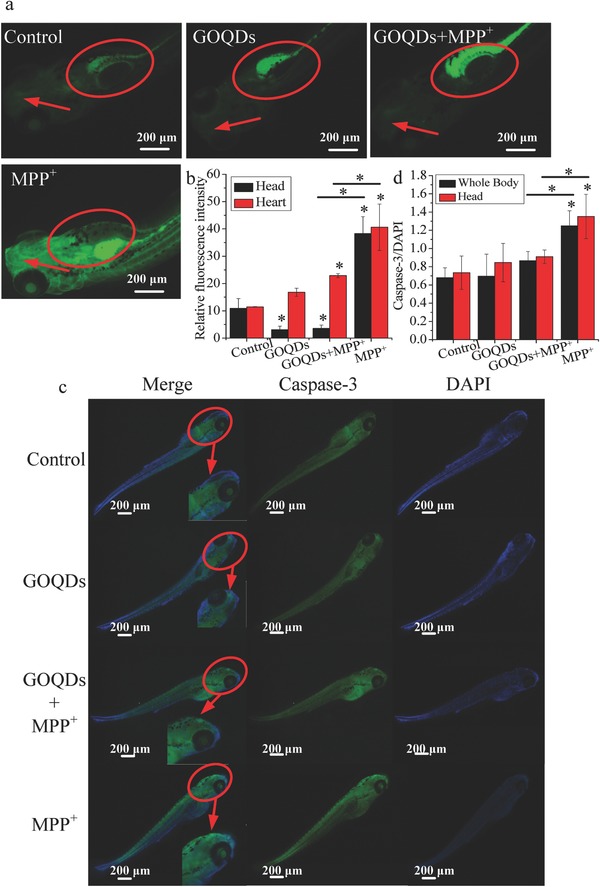
Effects of GOQDs on MPP^+^‐induced oxidative stress and apoptosis in vivo. a) Heads and hearts of larvae stained with DCFH‐DA. The heads and hearts are indicated by red arrows and circles, respectively. b) Quantitative analysis of the relative fluorescence intensity for ROS in the heads and hearts of the larvae. c) Caspase‐3 expression in zebrafish treated with MPP^+^ with or without preincubation with GOQDs by whole‐mount immunofluorescence staining. The heads of the larvae are denoted with red circles. The right bottom corner in each merged figure contains a magnified image of the circled area. d) Quantification of caspase‐3 levels in the whole body and head of larvae. *P < 0.05, compared with the control. * *P* < 0.05, GOQDs‐pretreated group compared with the MPP^+^‐treated group.

**Figure 7:**
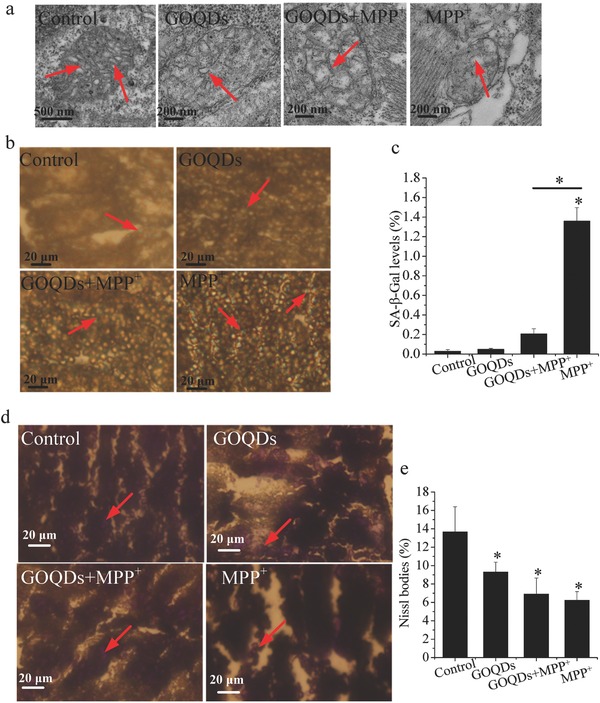
Effects of GOQDs on MPP^+^‐induced neuropathological responses in vivo. a) TEM images of mitochondria in the brain cells of zebrafish. Red arrows denote the internal crista structures of the mitochondria. b) Senescent cells stained with senescence‐associated β‐galactosidase in the ventral diencephalon of the larvae. The blue cells indicated by red arrows are senescent cells. c) Ratios of senescent cell areas to brain section areas. d) Nissl staining images of larvae brains. The purple cells denoted by red arrows are Nissl bodies. e) Quantification of Nissl bodies in the brains of the larvae. *P < 0.05, compared with the control. * *P* < 0.05, GOQDs‐pretreated group compared with the MPP^+^‐treated group.

Compared with the controls, MPP^+^ increased the ROS levels by 221.8% and 253% in the head and heart, respectively. In contrast, compared with the MPP^+^ group, the ROS levels decreased by 90.6% and 43.6% in the head and heart, respectively, after the GOQDs pretreatment (**Figure** **6**a,b). The caspase‐3 activities in both the head and whole body of the MPP^+^‐treated zebrafish were over 80% higher than those of the control (Figure 6c,d). In the group pretreated with GOQDs, the caspase‐3 activities decreased by 30.4%–32.6% compared with the MPP^+^‐treated group (Figure 6c,d), suggesting that apoptosis induced by MPP^+^ was mitigated by GOQDs. Ultramicroscopic structural analysis by TEM demonstrated that MPP^+^ induced mitochondrial lesions, damage to the mitochondrial outer membrane and loss of cristae (**Figure** **7**a). However, the mitochondria exhibited normal morphology with intact membrane and cristae structures when pretreated with the GOQDs under MPP^+^ exposure (Figure 7a). In the control and MPP^+^‐treated groups, the ratios of senescent cell areas to the brain areas were approximately 0.03% and 1.36%, respectively (Figure 7b,c). In contrast, the percentage of SA‐β‐Gal expression in the brains of the GOQDs‐pretreated zebrafish was significantly reduced to 0.21% under the MPP^+^ treatment (Figure 7b,c). As a characteristic neural structure, the number of Nissl bodies reflects the state of neurons.^20^ In physiological conditions, the Nissl bodies were large and abundant in the control and GOQDs‐treated groups, showing that the function of neuronal protein synthesis was strong (Figure 7d,e). The Nissl bodies were lightly stained and appeared to be sparsely arranged in the ventral diencephalon of the zebrafish treated with MPP^+^. The Nissl bodies in the ventral diencephalon of the GOQDs‐pretreated zebrafish increased by 11.3% even though no significance was evident compared to the MPP^+^‐treated group (Figure 7d,e). Based on the results of other indicators such as ROS, apoptosis, and senescent cells, significant differences were evident between the GOQDs‐pretreated and MPP^+^‐treated groups. Taken together, these observations indicated that the GOQDs could alleviate neuronal damage induced by MPP^+^.

### 2.8 Metabolomics Analysis in the Zebrafish Brain

The relative abundances of the metabolites in the brains of the zebrafish are presented in heat maps (Figure S8a, Supporting Information). HCL analysis demonstrated that MPP^+^ affected the metabolic profiles, but the alterations were mitigated by the GOQDs pretreatment. The differences among all tested groups (Figure S8b, Supporting Information) and the PCA scores plot (Figure S8c, Supporting Information) also confirmed that the metabolic disturbance in the brain of the larval zebrafish induced by MPP^+^ was mitigated by the GOQDs pretreatment. Moreover, the associations between metabolism and ROS were analyzed using the PLS model with ROS as the *Y* variable and the metabolic levels as the *X* variables (Figure S8d, Supporting Information). In addition, the correlations between the changes in pathophysiological indicators (malformation rate, ROS levels, locomotive activity, caspase‐3, SA‐β‐Gal, and Nissl bodies) and the corresponding two metabolites with the largest VIP values were analyzed by linear fitting. *R* ^2^ was from 0.85 to 0.97, which showed that the pathophysiological changes and metabolite regulation were closely related (Figure S9, Supporting Information). For example, Nissl bodies were negatively correlated with putrescine (*R* ^2^ = 0.95) and cadaverine (*R* ^2^ = 0.85), which was consistent with reports showing that the concentrations of putrescine and cadaverine were increased in patients with Alzheimer's disease and Parkinson's disease.^21^ The above results indicated that the metabolites were responsible for neurotoxicity and that their regulation by GOQDs was protective in vivo.

## 3 Discussion

ROS such as H_2_O_2_ and •OH are highly reactive and can modify intracellular molecules, disrupt the redox balance, and subject the cells to oxidative stress,^22^ which induces various biological responses, including neurotoxicity.^23^ Our recent study showed that GO nanosheets translocated into the brains of zebrafish and induced oxidative stress and neurotoxicity.[[qv: 5b]] Overproduction of ROS induced by MPP^+^ was mitigated by pretreatment with GOQDs in PC12 cells and the brains of larval zebrafish. The apoptosis, mitochondrial damage, and senescence induced by MPP^+^ were also mitigated by pretreatment with GOQDs. In addition, previous reports have shown that senescence is accompanied by a decline in behavioral functions,[[qv: 3c,23d]] which is a defining characteristic of neurotoxicity.[[qv: 5b,24]] Behavioral impairments in larval zebrafish were mitigated by pretreatment with GOQDs, suggesting that GOQDs provided neuroprotection. Experimental data have also shown aggregation of α‐synuclein in MPP^+^‐induced cells, which is a major component of Lewy bodies, the pathological hallmark of neurodegeneration.^25^ Pretreatment with GOQDs reduced the expression of α‐synuclein, further confirming that the GOQDs mitigated neurotoxicity. Meanwhile, the major changes in the Nissl bodies, including the dissolution and disappearance of the Nissl bodies, were associated with neuronal injury.^20^ Nissl body expression increased in the GOQDs‐pretreated group, resulting in an increase in the healthy neurons observed in the zebrafish brains. GOQDs reduced the H_2_O_2_ and ROS overproduction induced by MPP^+^ in PC12 cells and larval zebrafish by translocating into the cells and brains,^26^ which suggested that the GOQDs retained their catalase‐like activity in the intracellular environment. The catalase‐like activity probably contributed to the protective roles of the GOQDs. Recently, the intrinsic catalase mimetic activity of iron oxide nanoparticles, vanadium oxides nanoflakes, and gold nanoclusters has also been identified.[[qv: 3c,27]]

**Figure 8:**
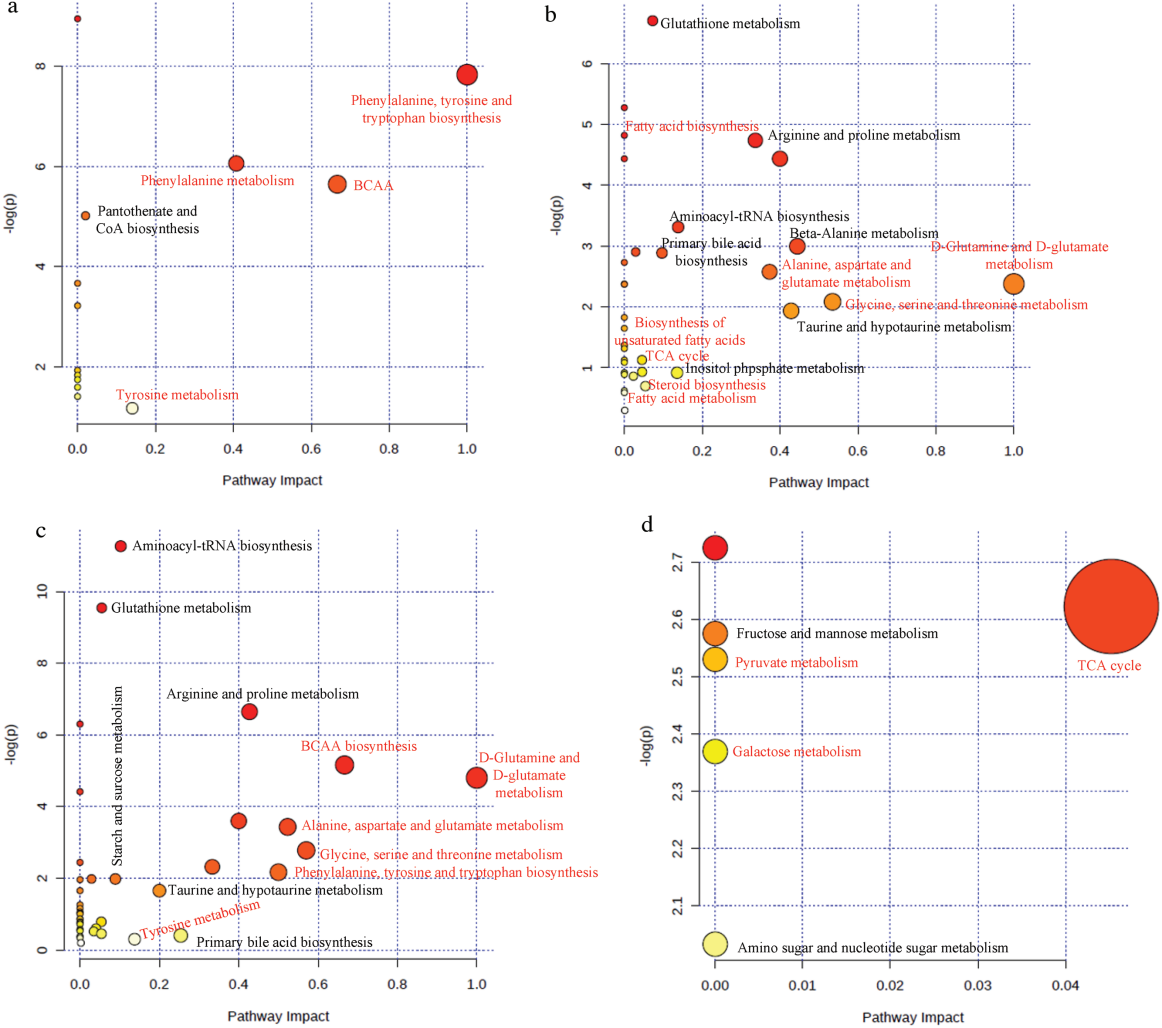
Overview of the altered metabolic profiles in vitro and in vivo in the GOQDs‐pretreated group compared to the MPP^+^‐treated group. a) Upregulation of metabolic pathways in vitro. b) Downregulation of metabolic pathways in vitro. c) Upregulation of metabolic pathways in vivo. d) Downregulation of metabolic pathways in vivo. The metabolic pathways in red are major pathways. BCAA: branched‐chain amino acid (valine, leucine, and isoleucine).

Phenylalanine influences the biosynthesis of serotonin, which is an important neurotransmitter.^28^ Tyrosine can be converted to L‐dopa, which is the precursor of dopamine and is associated with locomotive activity.^29^ Branched‐chain amino acids (BCAAs) directly and indirectly participate in protein synthesis, tissue‐defect repair, and energy production in the brain.^30^ Phenylalanine, tyrosine and tryptophan biosynthesis, tyrosine metabolism, and BCAAs (valine, leucine, and isoleucine) were enhanced by pretreatment with GOQDs (**Figure** **8**a,c). Furthermore, multiple amino acids, such as alanine, aspartate, glutamate, proline, glycine, serine, and taurine, serve as neurotransmitters, neuromodulators, and antioxidants.^31^ These amino acids were increased in the brains of the zebrafish in the GOQDs‐pretreated group compared with the MPP^+^‐treated group (Figure 8c). The tricarboxylic acid (TCA) cycle comprises a cascade of oxidative reactions that generate energy in the mitochondria.^32^ The TCA cycle was inhibited by the pretreatment with GOQDs compared to the treatment with MPP^+^ (Figure 8b,d). ROS significantly damages the central nervous system (CNS) due to the high content of unsaturated fatty acids, which are susceptible to peroxidation, in the CNS.^33^ Cholesterol is also very sensitive to oxidative stress.^34^ Therefore, the decrease in cholesterol and fatty acids mediated by GOQDs inhibited the ROS attack against the CNS (Figure 8b). Chronic administration of galactose induces oxidative damage and neurogenesis in rodent brains.^35^ In this study, galactose metabolism was decreased by the pretreatment with GOQDs (Figure 8d), which may promote neurogenesis in zebrafish.

## 4 Conclusions

Our recent study showed that GO nanosheets translocated into the brains of zebrafish and induced oxidative stress and neurotoxicity.[[qv: 5b]] Compared with GO, GOQDs protected PC12 cells and larval zebrafish from neurotoxicity; the underlying mechanisms are shown in Figure S10 (Supporting Information). In vitro, the GOQDs efficiently reduced MPP^+^‐induced ROS, H_2_O_2_, cell toxicity, apoptosis, and SA‐β‐Gal expression. The GOQDs protected against neurotoxicity by inhibiting the expression of α‐synuclein. In vivo, the GOQDs efficiently diminished MPP^+^‐induced ROS, mortality, the malformation rate, apoptosis, mitochondrial damage, and SA‐β‐Gal expression. Increased locomotive activity and Nissl bodies were observed in the brains of GOQDs‐pretreated larvae and further confirmed that the GOQDs mitigated neurotoxicity. The enhancement of amino acid metabolism and inhibition of the TCA cycle, steroid biosynthesis, fatty acid biosynthesis, and galactose metabolism by GOQDs, compared with MPP^+^, may participate in fundamental metabolic pathways related to antioxidation and neurotransmission and contribute to neurotoxic amelioration. Meanwhile, the decomposition of H_2_O_2_ and Fenton reactions implied the catalase‐like activity of the GOQDs. The present study indicates that GOQDs can mitigate neurotoxicity in vitro and in vivo via antioxidative activities and metabolic regulation.

## 5 Experimental Section

Characterization of GOQDs is described in the Supporting Information.

*Catalase‐Like Activity Analysis and Electron Paramagnetic Resonance (EPR) Measurements In Vitro*: The catalase‐like activity assay was carried out by adding 10 m H_2_O_2_ into 100 µg mL^−1^ GOQDs or 4 U mL^−1^ catalase. After 10 min, gas bubbles were observed. The catalase‐like activity of the GOQDs was also determined by assessing the rate of reduction of the H_2_O_2_ levels. The initial concentration of H_2_O_2_ was 25 mmol L^−1^. After decomposition by GOQDs or catalase, the final concentrations of H_2_O_2_ were detected with a catalase assay kit (Nanjing Jiancheng Bioengineering Institute, China) and measured at 405 nm using a microplate reader (BioTek H4 MLFA, USA). With 5,5‐dimethyl‐1‐pyrroline N‐oxide as a probe. The OH levels in the Fenton reactions and GOQDs or catalase reactions were detected using the EPR (Magnettech MiniScope 400, Germany) with MiniScope Control software as described previously.^14^

*Synthesis of FITC‐GOQDs and Analysis of the Translocation of GOQDs*: FITC‐GOQDs synthesis was performed as described previously.^15^ Briefly, 100 µg mL^−1^ GOQDs and 1 µg mL^−1^ FITC were mixed at a 100:1 ratio under sonication for 30 min in ice water. Then, the mixture was filtered twice through a 30 kDa MW spin filter (Vivaspin, Sartorius, Germany) at 3000 g to remove free FITC. For confirmation of GOQDs labeling with FITC, the UV–vis spectra of the FITC‐GOQDs and FITC were determined on a T90 spectrophotometer (Purkinje General, Beijing, China). Larval zebrafish at 24 hpf (with chorion) and 72 hpf (hatched from chorion) were incubated with E3 medium, FITC‐GOQDs, FITC, or GOQDs. After 30 min at 28 °C, larval zebrafish were observed by laser scanning confocal microscopy (LSCM; Leica, TCS SP8, Wetzlar, Germany).

*ROS and H_2_O_2_ Levels*: Detailed descriptions of the PC12 cell culture, zebrafish maintenance, measurement of cell viability, and assessment of mortality and malformation in larval zebrafish are provided in the Supporting Information. All animals used in the experiments were obtained from China Zebrafish Resource Center (CZRC), and the care provided to the animals used in the experiments complied with institutional guidelines for the health and care of experimental animals. The Committee on the Ethics of Animal Experiments of Nankai University approved these protocols. ROS generation in vitro and in vivo was measured with the 2′,7′‐dichlorofluorescein diacetate (DCFH‐DA) method. Fluorescence was observed via LSCM (Leica, TCS SP8, Germany) and an inverted fluorescence microscope (Olympus X71; Olympus, Japan) with an excitation wavelength of 485 nm and an emission wavelength of 535 nm. The relative fluorescence intensity was quantified with ImageJ software (NIH, USA). The intracellular H_2_O_2_ levels were detected with a Hydrogen Peroxide Colorimetric/Fluorometric Assay Kit (Biovision, USA). The absorbance of each sample was measured at 570 nm using a microplate reader (BioTek H4 MLFA, USA).

*SA‐β‐Gal and Nissl Staining*: PC12 cells were stained with SA‐β‐Gal kit (Beyotime, China). Larval zebrafish at 120 hpf were anesthetized with tricaine (TCI, Shanghai, China), and the brain tissues were harvested by scalpel and immediately fixed in 4% paraformaldehyde. The tissues were processed routinely for paraffin embedding, and 8 µm thick sections were cut and mounted onto glass slides. The tissue samples were stained with SA‐β‐Gal or Nissl solution (Beyotime, China). The samples were evaluated and photographed using a microscope (OlympusX71, Olympus, Japan). Senescence levels and Nissl bodies were quantified with Photoshop 13.0 (Adobe, USA) and ImageJ software (NIH, USA).

*TEM Imaging*: At the end of the exposure period, 1 × 10^6^ PC12 cells or eight larval zebrafish in each group were selected for TEM imaging as described in a previous study.[[qv: 5b]] Briefly, samples were fixed in 2.5% glutaric dialdehyde and then in 1% osmic acid for 2 h. After dehydration, the specimens were embedded in Epon. Ultrathin sections were prepared using an ultramicrotome (Leica EM UC7&FC7, Germany) and stained with uranyl acetate and lead citrate. Finally, the sections were examined using high‐resolution TEM (Hitachi HT7700, Japan).

*Western Blotting*: Western blotting was performed as described previously.^36^ Briefly, 11 µg of protein from PC12 cells was separated on a 10% sodium dodecyl sulfate‐polyacrylamide gel. Next, proteins were transferred to nitrocellulose membranes and incubated with primary antibody for 2 h at 37 °C. Blots were washed for 4 × 10 min in Tris‐buffered saline with 0.1% Tween‐20 (pH 7.6). Then, the blots were incubated with 1:2000 HRP‐conjugated goat antirabbit IgG (Jackson ImmunoResearch Laboratories, Inc., USA) for 2 h at 37 °C and developed using Super‐GL ECL enhanced chemiluminescence substrates. The films were scanned, and then, grayscale analysis of the blots was performed using the Gel‐Pro Analyzer (Media Cybernetics, USA). The antibodies for α‐synuclein (1:250), caspase‐3 (1:1000), Bcl‐2 (1:1000) ,and Bax (1:3000) detection were obtained from Abcam, UK. The antiglyceraldehyde‐3‐phosphate dehydrogenase (GAPDH) antibody (1:10 000) (Abcam, UK) was employed as an internal standard to monitor loading errors.

*Whole‐Mount IF Staining*: Larval zebrafish at 120 hpf were anesthetized with tricaine, washed in phosphate‐buffered saline three times and fixed with 4% paraformaldehyde at room temperature. Samples were incubated with anticleaved caspase‐3 polyclonal antibody (Cell Signaling Technology, USA) and Alexa Fluor 488‐conjugated secondary antibody (Cell Signaling Technology, USA), followed by 4,6‐diamino‐2‐phenyl indole (Sigma, USA) staining and fluorescence imaging by fluorescence microscopy (Olympus X71, Olympus, Japan). The relative fluorescence intensity was quantified with ImageJ software (NIH, USA).

*Behavior Analysis*: Larval zebrafish at 7 d postfertilization (dpf) were pipetted into a 96‐well plate. A camera (Canon EOS 700D, Japan) was fixed above the plate for vertical observations. The fish were allowed to acclimate for 5 min, and then, swimming videos were recorded for 5 min. The total swimming distance and swim velocity (total swimming distance divided by the duration of swimming movement) were analyzed by ZebraLab 3.3 software (ViewPoint, France). Twelve zebrafish were recorded for each replicate.

*Metabolomics Analysis*: Metabolomics analysis was performed as described previously.[[qv: 5b]] Briefly, 1 × 10^6^ PC12 cells and 80 larval zebrafish brains at 120 hpf were collected and rapidly frozen in liquid nitrogen. Samples were homogenized in 2 mL of cold methanol–chloroform–ultrapure water (volume ratio, 2.5:1:1). Homogenate was extracted twice using a microwave (MDS‐8, SINEO, China) at 40 °C for 20 min. After centrifugation, the supernatants were mixed, and the mixture was dried under nitrogen gas and vacuum freeze‐dried. Derivatization of the sample was performed using 50 mL of O‐methyl hydroxyl amine hydrochloride pyridine (20 mg mL^−1^) at 30 °C for 90 min, followed by derivatization using 80 mL of N‐methyl‐N‐(trimethylsilyl) trifluoroacetamide at 37 °C for 30 min. The samples were centrifuged at 10 000 g for 5 min, and the supernatants were analyzed using gas chromatography‐mass spectrometry (Agilent 6890N‐5973, USA).

*Statistical Analysis*: The measurement for each treatment was repeated in triplicate unless otherwise noted. The error bars represent the standard deviation. Statistical significance was determined using analysis of variance with SPSS 22.0. Differences were considered statistically significant when the *P*‐value was less than 0.05. PCA and PLS were performed using SIMCA‐P 13.0 software. The default distance metric for HCL was Pearson's correlation, and the linkage method selection was determined using the average linkage clustering.

## Conflict of Interest

The authors declare no conflict of interest.

## Supporting information

Supplementary
