# Supplementary material for: Graphene Oxide Quantum Dots Reduce Oxidative Stress and Inhibit Neurotoxicity In Vitro and In Vivo through Catalase‐Like Activity and Metabolic Regulation
Source: Adv Sci (Weinh). 2018 Mar 4;5(5):1700595. doi: 10.1002/advs.201700595 (PMC5978962; doi:10.1002/advs.201700595)
Supplement: Supplementary file 1 — Supplementary [file ADVS-5-1700595-s001.pdf]

Copyright WILEY-VCH Verlag GmbH & Co. KGaA, 69469 Weinheim, Germany, 2018.

Supporting Information

for *Adv. Sci.,* DOI: 10.1002/advs.201700595

Graphene Oxide Quantum Dots Reduce Oxidative Stress and Inhibit Neurotoxicity In Vitro and In Vivo through Catalase-Like Activity and Metabolic Regulation

*Chaoxiu Ren, Xiangang Hu, and Qixing Zhou**

Supporting Information

Graphene Oxide Quantum Dots Reduce Oxidative Stress and Inhibit Neurotoxicity *in vitro* and *in vivo* through Catalase-like Activity and Metabolic Regulation

Chaoxiu Ren, Qixing Zhou, Xiangang Hu

Key Laboratory of Pollution Processes and Environmental Criteria (Ministry of Education)/Tianjin Key Laboratory of Environmental Remediation and Pollution Control, College of Environmental Science and Engineering, Nankai University, Tianjin 300071, China.

Corresponding author: Qixing Zhou (qxzhou523@163.com),

Xiangang Hu (huxiangang@nankai.edu.cn).

Fax, 0086-022-85358121; Tel, 0086-022-85358121.

Page number: 14

Figure number:10

Experimental Section

Cell culture and zebrafish maintenance: PC12 cells (Dingguo Changsheng Biotechnology CO., LTD., China) were maintained in Dulbecco's modified Eagle's medium (high glucose) (DMEM-H, GENVIEW, USA) with 10% fetal bovine serum (FBS) at 37°C and 5% CO_2 (BPN-240RHP, Yi Heng Scientific Instrument Co., Ltd., China). Zebrafish (wild-type AB strain, 6 month) were maintained at 28°C with a 14:10 h light/dark cycle and fed living brine shrimp twice daily. A continuous water cycling system was used to maintain water quality. Embryos were collected by natural spawning and raised in E3 solution (5 mM NaCl, 0.17 mM KCl, 0.33 mM $CaCl_2$, 0.33 mM $MgSO_4$, pH 7.4). All embryos were incubated at 28°C in a climate-controlled cabinet (SPX-300I-C, BOXUN, China) during development.

Characteristics of graphene oxide quantum dots (GOQDs): GOQDs were purchased from XFNANO (XF042, China). Atomic force microscopy (AFM) and HRTEM, Hitachi HT7700, Japan) were conducted on a Nanoscope 4 (Veeco, USA) and a JEM-2010 FEF (JEOL, Japan), respectively. The hydrodynamic diameters (Hds) of the GOQDs in E3 medium and the cell culture medium were detected to evaluate the size distribution of the GOQDs with a Zetasizer Nano ZS90 (Malvern, UK).

Cell toxicity assays and cell morphology: PC12 cells were treated with 1-methyl-4-phenyl-pyridinium ion (MPP^+, Sigma, USA) for 24 h in with or without preincubation with GOQDs. Cells were incubated with cell culture medium (control) or GOQDs alone for 24 h. Cell viability was measured with Cell Counting Kit-8 (CCK-8, Beyotime, China) as described previously.[1] The absorbance of each well was measured at 450 nm using a microplate reader (BioTek H4 MLFA, USA). The cells in the control group had a good growth status, as shown in Figure 1b. The absorbance of the control group was set as 100% cell viability, and the absorbance of the treated group divided by that of the control group indicated the cell viability of the treated group.

Three replicate wells were used for each sample and the control. The living cell morphology images were obtained with an inverted microscope (OlympusX71, Olympus, Japan).

Mortality and malformation in larval zebrafish: Larval zebrafish were preincubated with GOQDs (100 μg mL^{-1}) from 6 h post-fertilization (hpf) to 72 hpf and then treated with MPP^+ (1.5 mM) until 120 hpf. Meanwhile, the groups of larval zebrafish were treated with E3 medium (control), GOQDs or MPP^+. The mortality and malformation rates and images of the larval zebrafish were recorded at 120 hpf by light microscopy (Olympus ZL 61, Olympus, Japan).

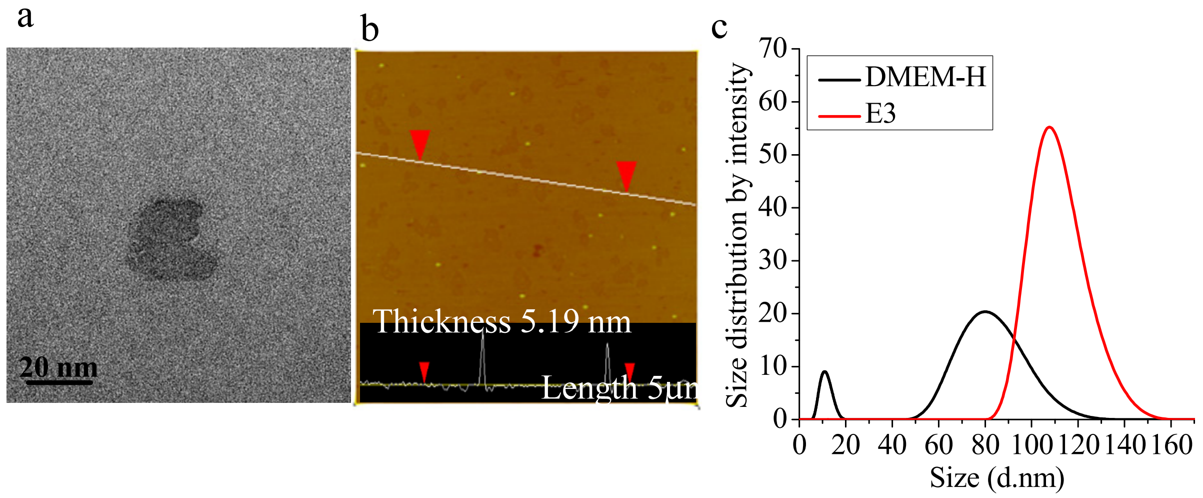

Figure S1 Characteristics of nanomaterials. a) TEM imaging. b) AFM imaging. c) Size distribution of the GOQDs in complete DMEM-H with 10% FBS (black line) and in E3 medium (red line).

The lateral sizes and thicknesses of the GOQDs were determined by TEM and AFM (Figure S1a and S1b). The lateral sizes ranged from approximately 20 to 40 nm. The thicknesses of the GOQDs ranged from 4.18 to 5.19 nm. The size distribution (Figure S1c) revealed that the hydrodynamic diameters of the GOQDs were approximately 75 nm in DMEM-H with 10% FBS (complete medium) and approximately 105 nm in E3 medium. The small fractions with hydrodynamic

diameters from 6.5 to 18 nm were from the macromolecule compositions of the complete medium.

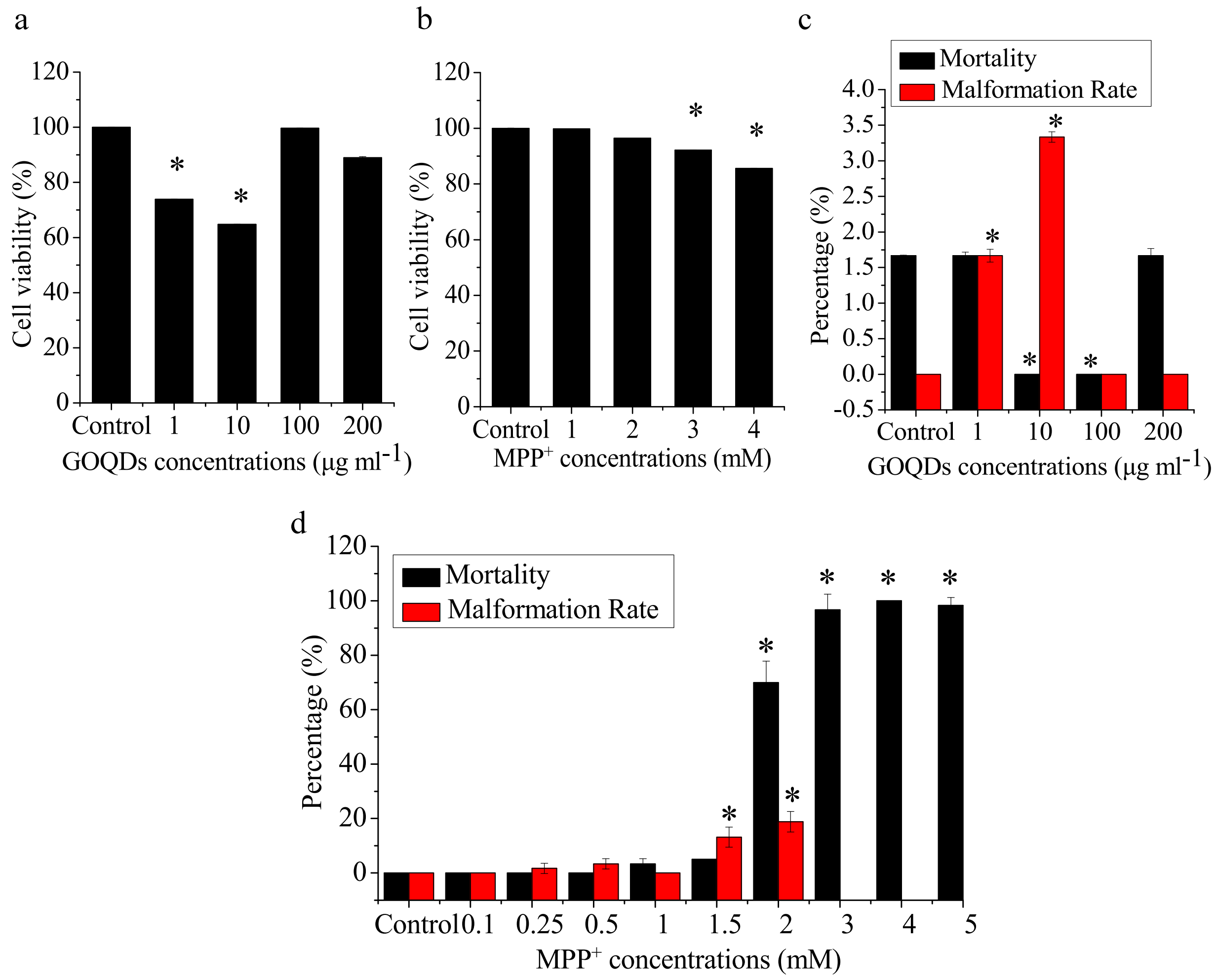

Figure S2 Effects of GOQDs and MPP^+ on PC12 cells and larval zebrafish. a) Cell viability of PC12 cells incubated with GOQDs. b) Cell viability of PC12 cells incubated with MPP^+. c) Mortality and malformation rates of larval zebrafish treated with GOQDs. d) Mortality and malformation rates of larval zebrafish treated with MPP^+. Measurement for each treatment was repeated in triplicate. The error bars were not obvious when the standard deviations were small.

GOQDs at 1 to 200 μg mL^{-1} were used to assess cell viability prior to use. At a concentration of 100 μg mL^{-1}, cell viability was 99.67% compared with 100% for the control, indicating that the

GOQDs were biocompatible with PC12 cells. In the subsequent experiments, GOQDs at 100 μg mL^{-1} were used for the treatments. Furthermore, cell viability decreased with increasing concentrations of MPP^+ from 1 to 4 mM and was significantly decreased by 15% at 4 mM MPP^+ (Figure S2b). In the present study, 4 mM MPP^+ was used to induce neurotoxicity in PC12 cells. Various concentrations of GOQDs and MPP^+ were examined to assess the mortality and malformation rates in larval zebrafish prior to the treatments with GOQDs (Figure S2c and S2d). The highest mortality and malformation rates of the zebrafish treated with GOQDs (1-200 μg mL^{-1}) were 1.7% and 3.3%, respectively. The results suggested that the GOQDs were biocompatible at the tested concentrations ranging from 1-200 μg mL^{-1}. In addition, Jasim et al. reported that glomerular excretion of significant amounts of GO did not induce any signs of acute nephrotoxicity or glomerular barrier dysfunction.[2] Therefore, GOQDs were still safe to use after biokinetic processing and excretion. In the present study, GOQDs at the biocompatible concentration of 100 μg mL^{-1} were used. The mortality rate of zebrafish treated with 2 mM MPP^+ was 70%, which was too high for further experiments, so 1.5 mM MPP^+ was chosen for the zebrafish experiments.

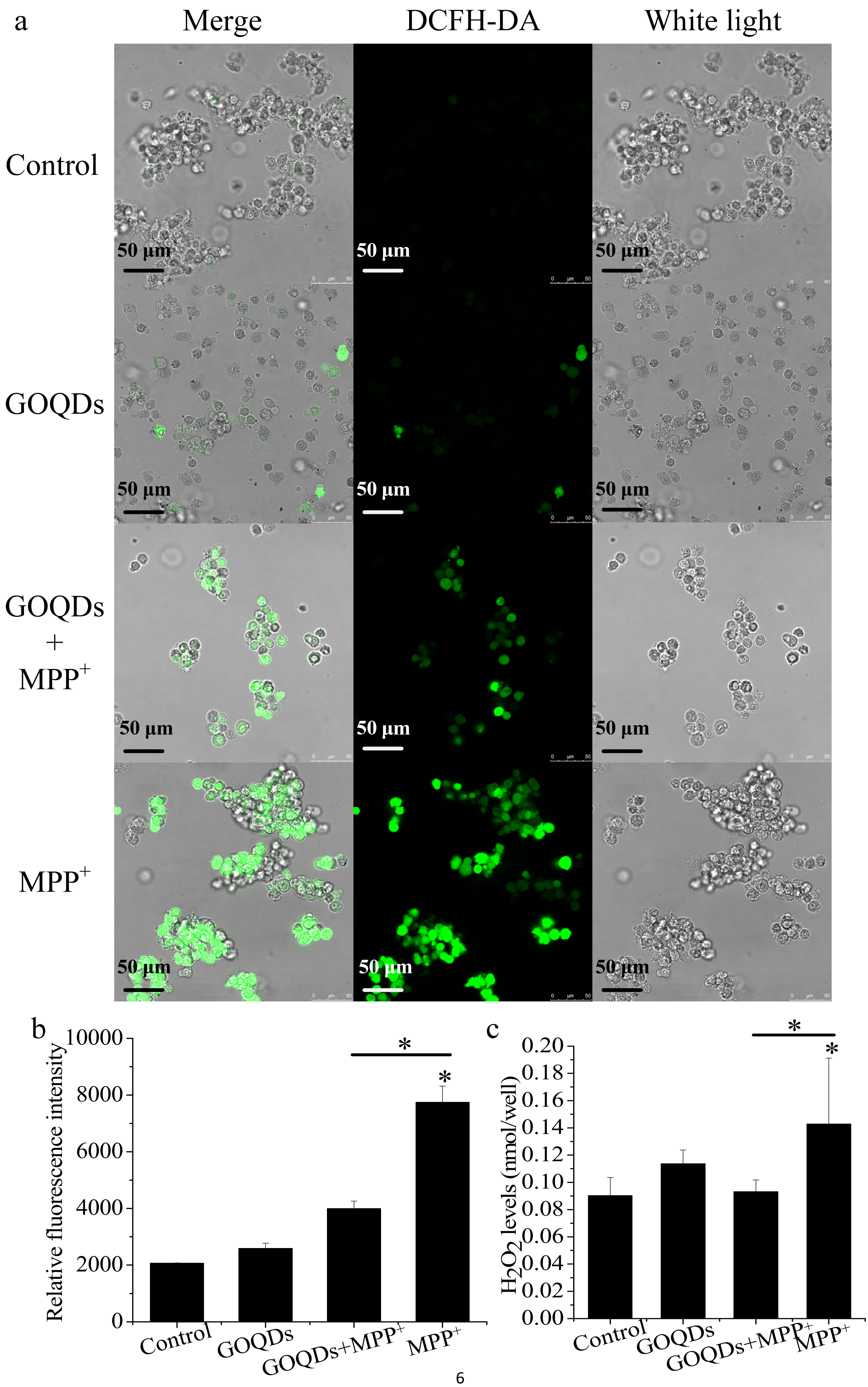
a
Merge
DCFH-DA
White light
Control
GOQDs
GOQDs
+
MPP+
MPP+
50 μm
b
Relative fluorescence intensity
0
2000
4000
6000
8000
10000
Control
GOQDs
GOQDs+MPP+
MPP+
*
*
c
H2O2 levels (nmol/well)
0.00
0.02
0.04
0.06
0.08
0.10
0.12
0.14
0.16
0.18
0.20
Control
GOQDs
GOQDs+MPP+
MPP+
*
*

88

Figure S3 Effects of GOQDs on oxidative stress induced by MPP^+. a) LSCM images of PC12 cells stained with 2′,7′-dichlorofluorescein diacetate (DCFH-DA). b) Quantification of ROS levels in PC12 cells. c) H_2O_2 levels in PC12 cells. **P*<0.05, compared with the control. **P*<0.05, GOQDs-pretreated group compared with the MPP^+-treated group.

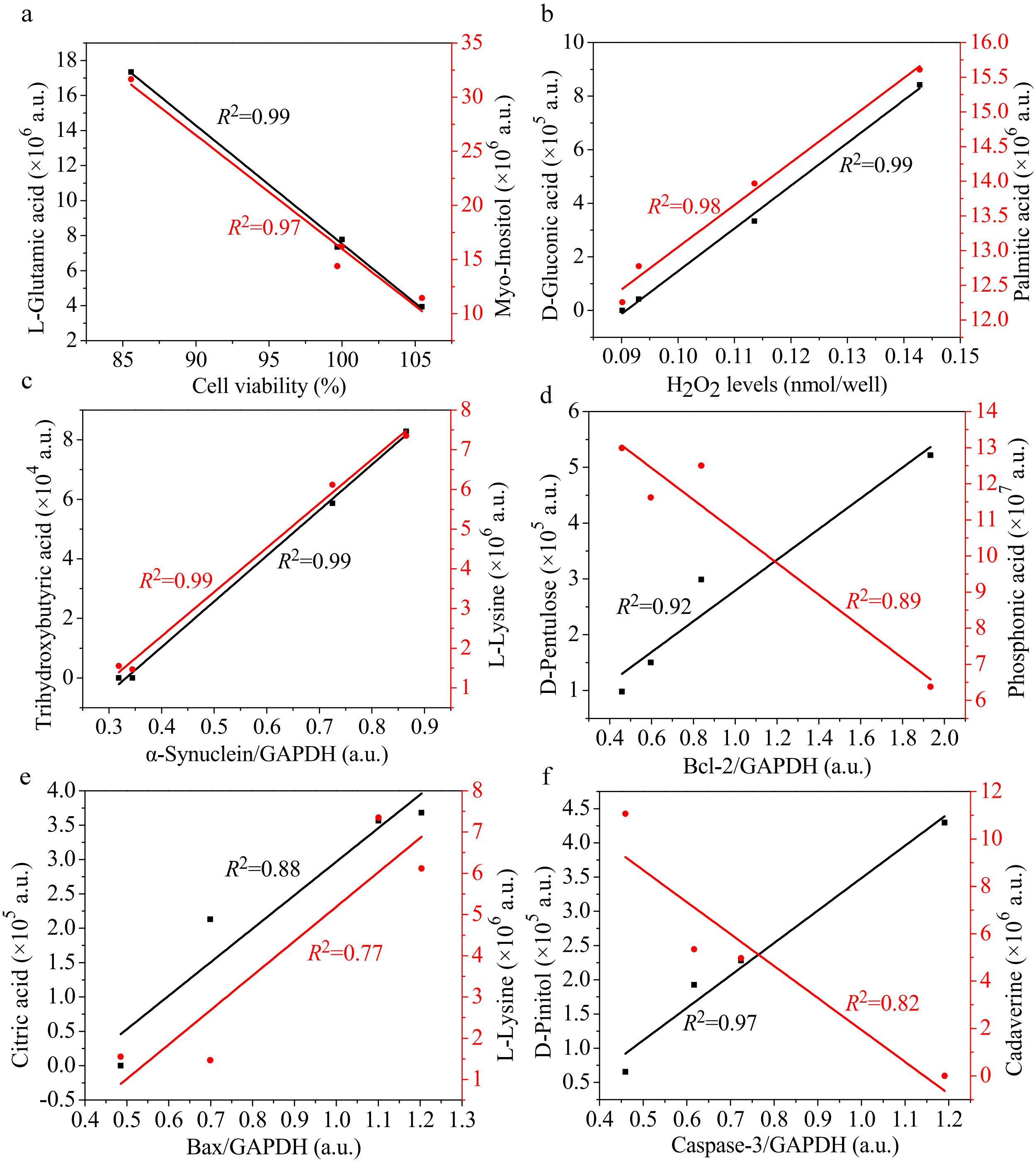

Figure S4 Correlation analysis of pathophysiological indicator changes and the corresponding two

metabolites with the largest VIP values by linear fitting *in vitro*. a) Correlation analysis of cell viability with L-glutamic acid and myo-inositol. b) Correlation analysis of H_2O_2 levels with D-gluconic acid and palmitic acid. c) Correlation analysis of α-synuclein with trihydroxybutyric acid and L-lysine. d) Correlation analysis of Bcl-2 with D-pentulose and phosphonic acid. e) Correlation analysis of Bax with citric acid and L-lysine. f) Correlation analysis of caspase-3 with D-pinitol and cadaverine.

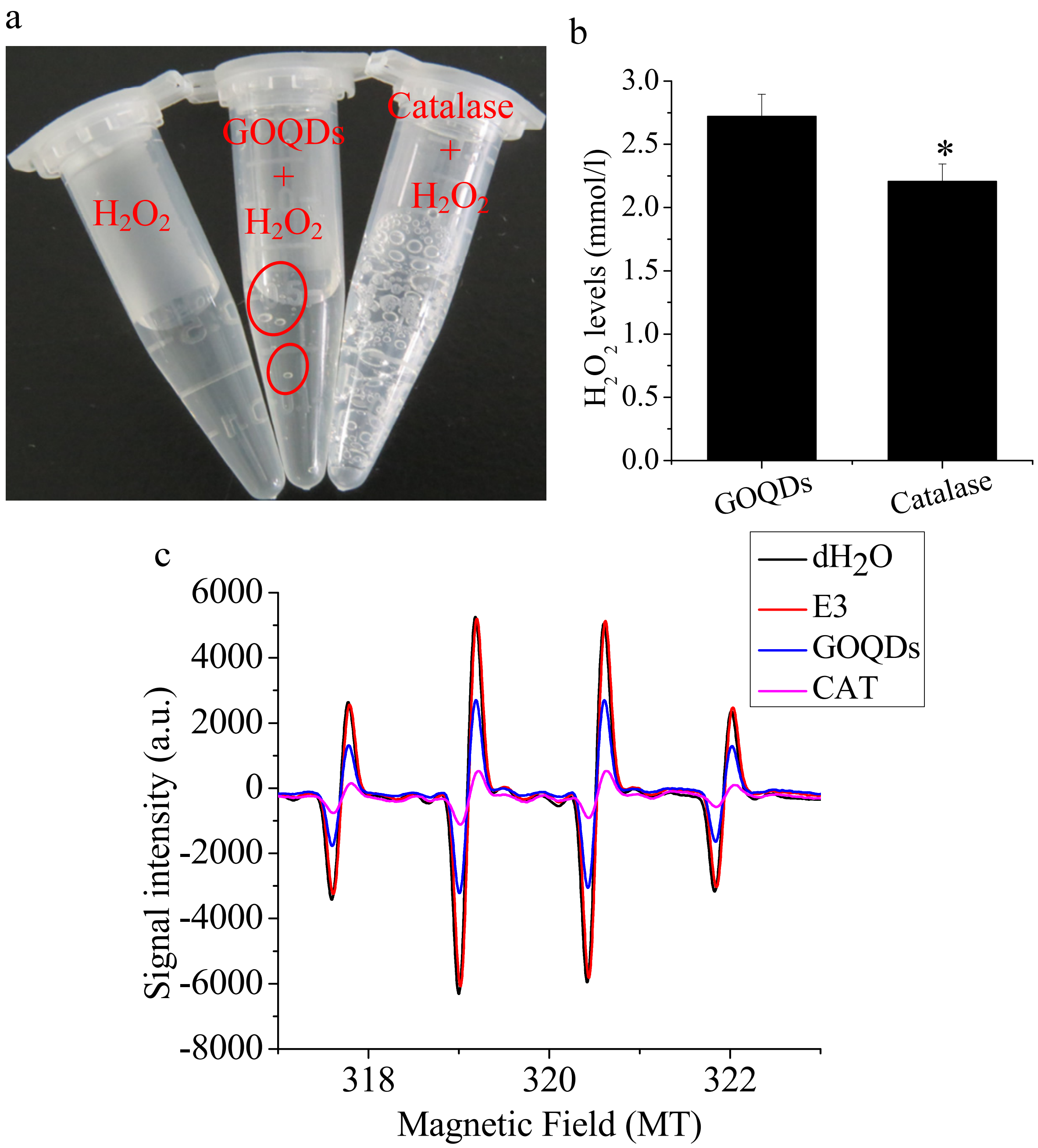

Figure S5 Catalase-like activity of GOQDs *in vitro*. a) Bubble production indicating the decomposition of H_2O_2 by GOQDs and catalase. The bubbles are circled in red in the GOQDs group. b) Relative levels of H_2O_2 after incubation with GOQDs (100 μg mL^{-1}) or catalase (4 U mL^{-1}). c) •OH signal intensity of Fenton reactions incubated with distilled water (dH_2O, black), E3 (red), GOQDs (blue) or catalase (CAT, pink). The Fenton reaction formulas were as follows: $Fe^{2+} + H_2O_2 \rightarrow Fe^{3+} + OH^- + \bullet OH$;[3] $H_2O_2 + Fe^{3+} \rightarrow Fe^{2+} + O_2 + 2H^+$; and $O_2 + Fe^{3+} \rightarrow Fe^{2+} + O_2 \cdot -$. **P*<0.05.

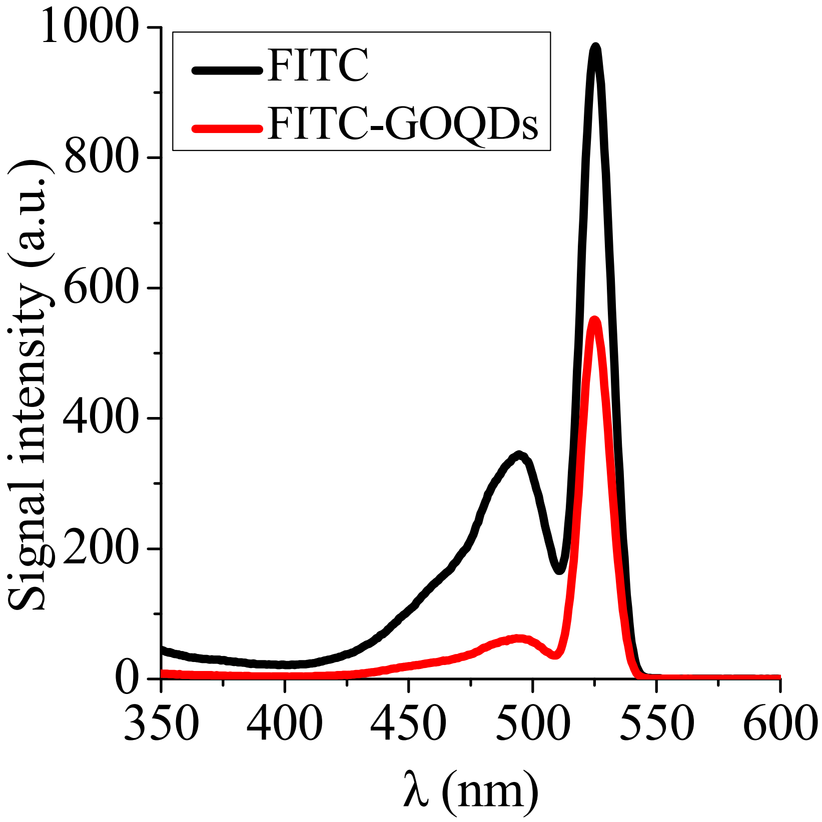

Figure S6 UV-vis absorption spectra of FITC (black) and FITC-GOQDs (red).

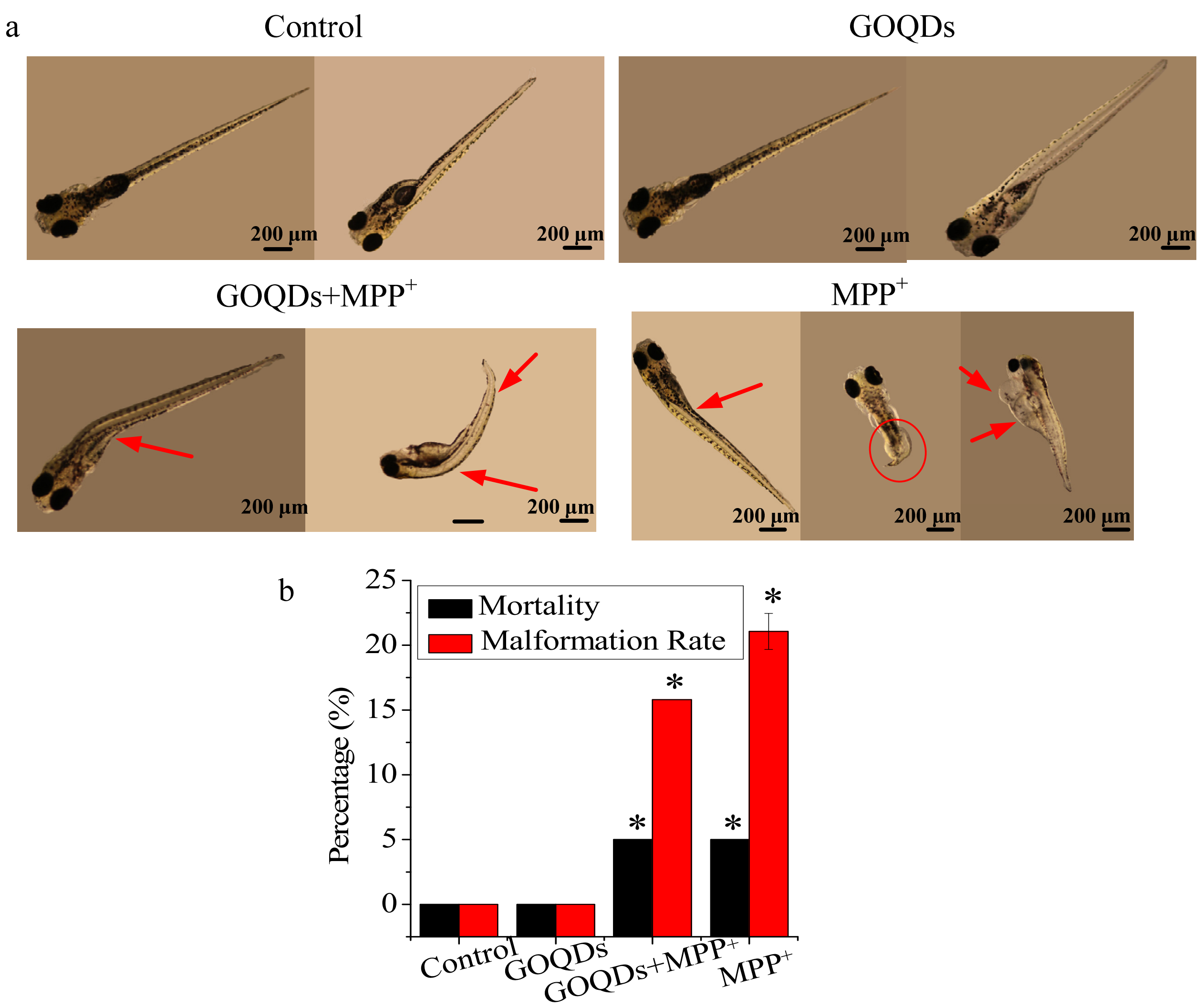

Figure S7 Effects of GOQDs on the MPP$^+$-associated mortality and malformation rates *in vivo*. a) Malformation in larvae treated with MPP$^+$ with or without preincubation with GOQDs. Tail/spinal curvature, rumplessness and pericardial/yolk sac edema are denoted by red arrows or circles. b) Quantification of the mortality and malformation rates. *P<0.05, compared with the control.

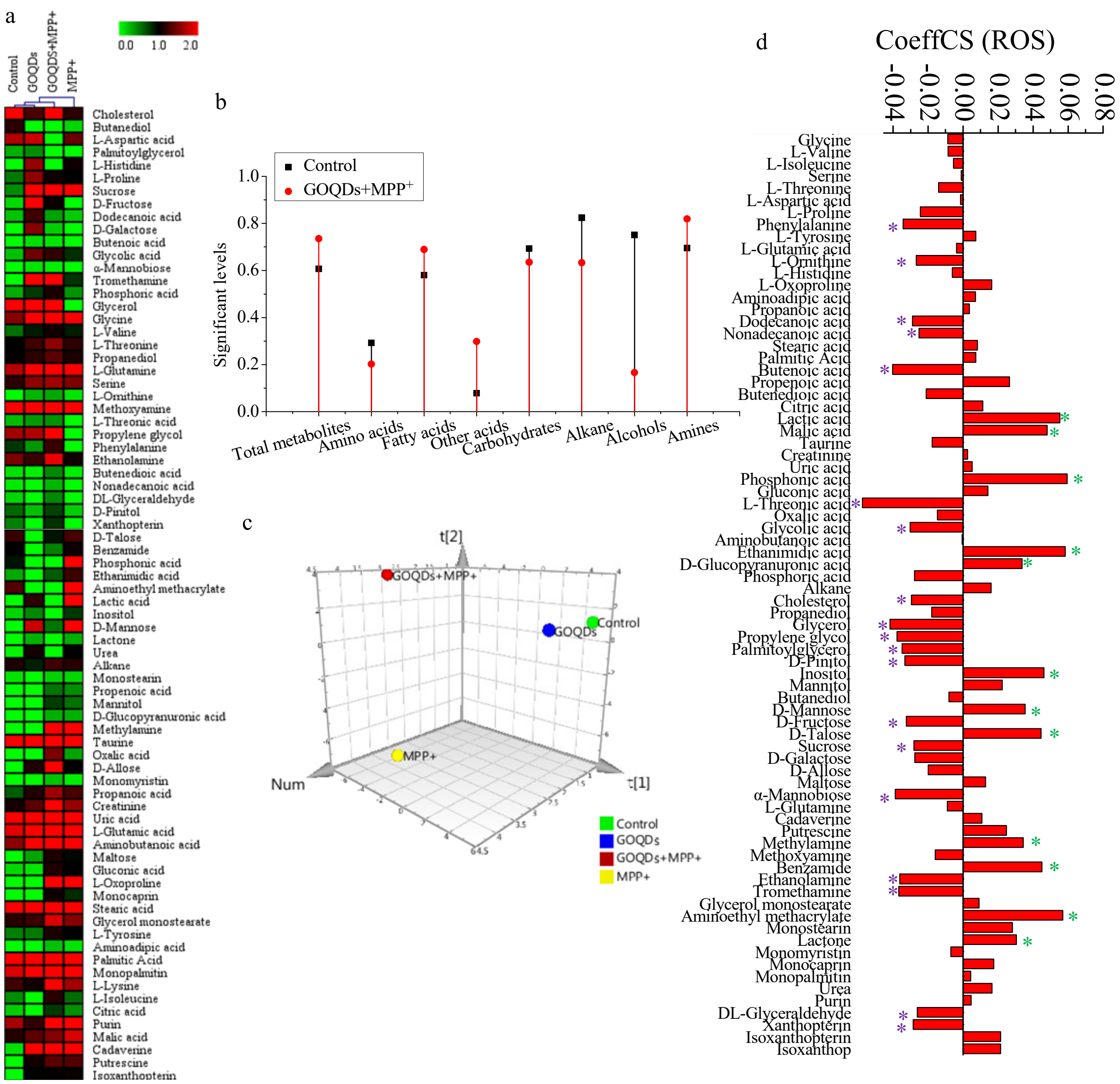

Figure S8 Effects of GOQDs on MPP^+-induced metabolomics in zebrafish brains. a) Heat maps of identified metabolites. b) Significant levels of metabolites in the control and GOQDs+MPP^+ groups compared with the MPP^+ group. c) Metabolic cluster analysis using PCA scores plot. d) CoeffCS of metabolites as the X variable and ROS as the Y variable by PLS. The metabolites labeled with asterisks represent the metabolites with a VIP greater than one. The metabolites labeled with green

and purple asterisks represent the metabolites that positively and negatively contribute to ROS, respectively.

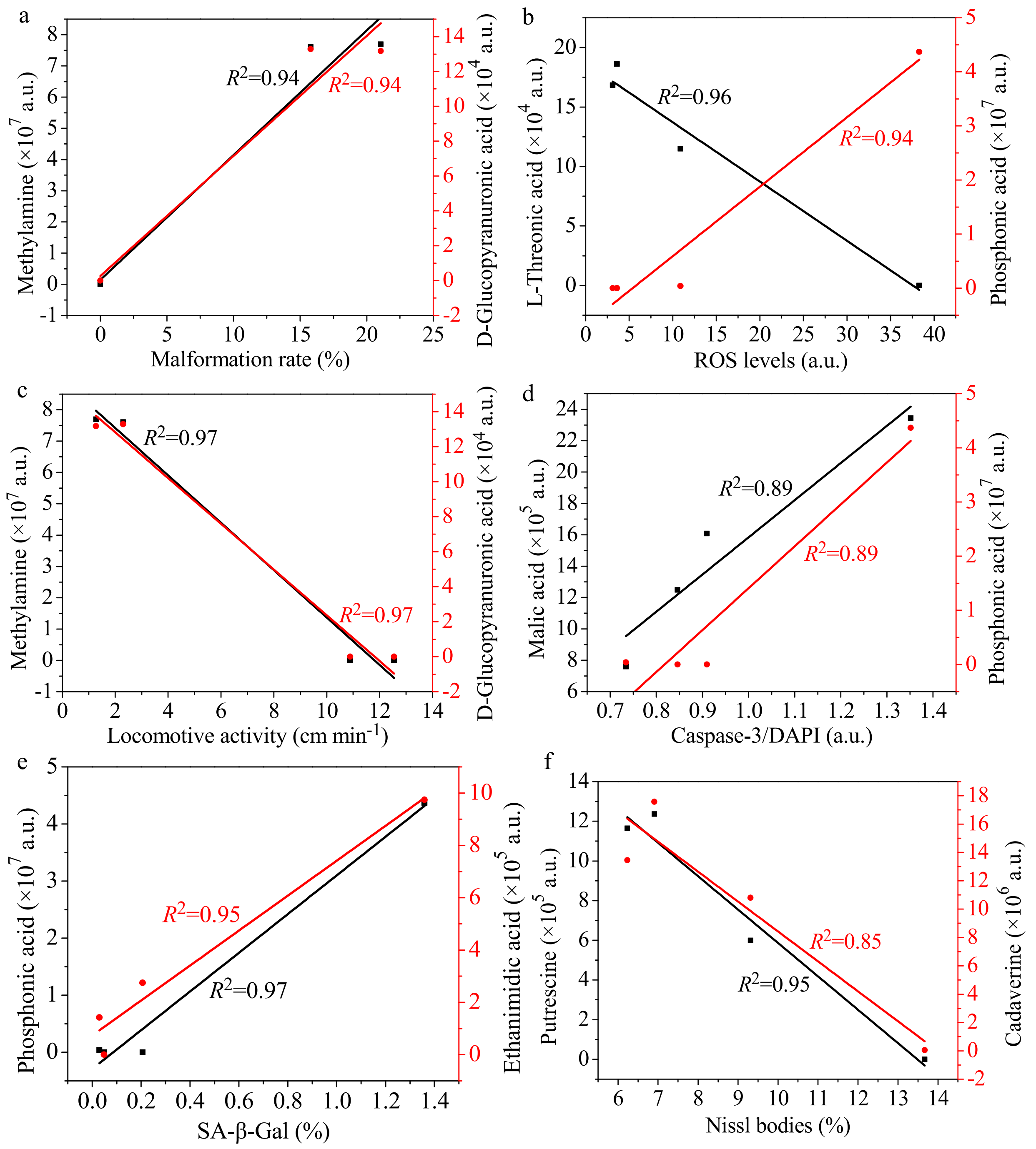

Figure S9. Correlation analysis of pathophysiological indicator changes and the corresponding two metabolites with the largest VIP values by linear fitting *in vivo*. a) Correlation analysis of the malformation rate with methylamine and D-glucopyranuronic acid. b) Correlation analysis of ROS

levels with L-threonic acid and phosphonic acid. c) Correlation analysis of locomotive activity with methylamine and D-glucopyranuronic acid. d) Correlation analysis of caspase-3 with malic acid and phosphonic acid. e) Correlation analysis of SA-β-Gal with phosphonic acid and ethanimidic acid. (f) Correlation analysis of Nissl bodies with putrescine and cadaverine.

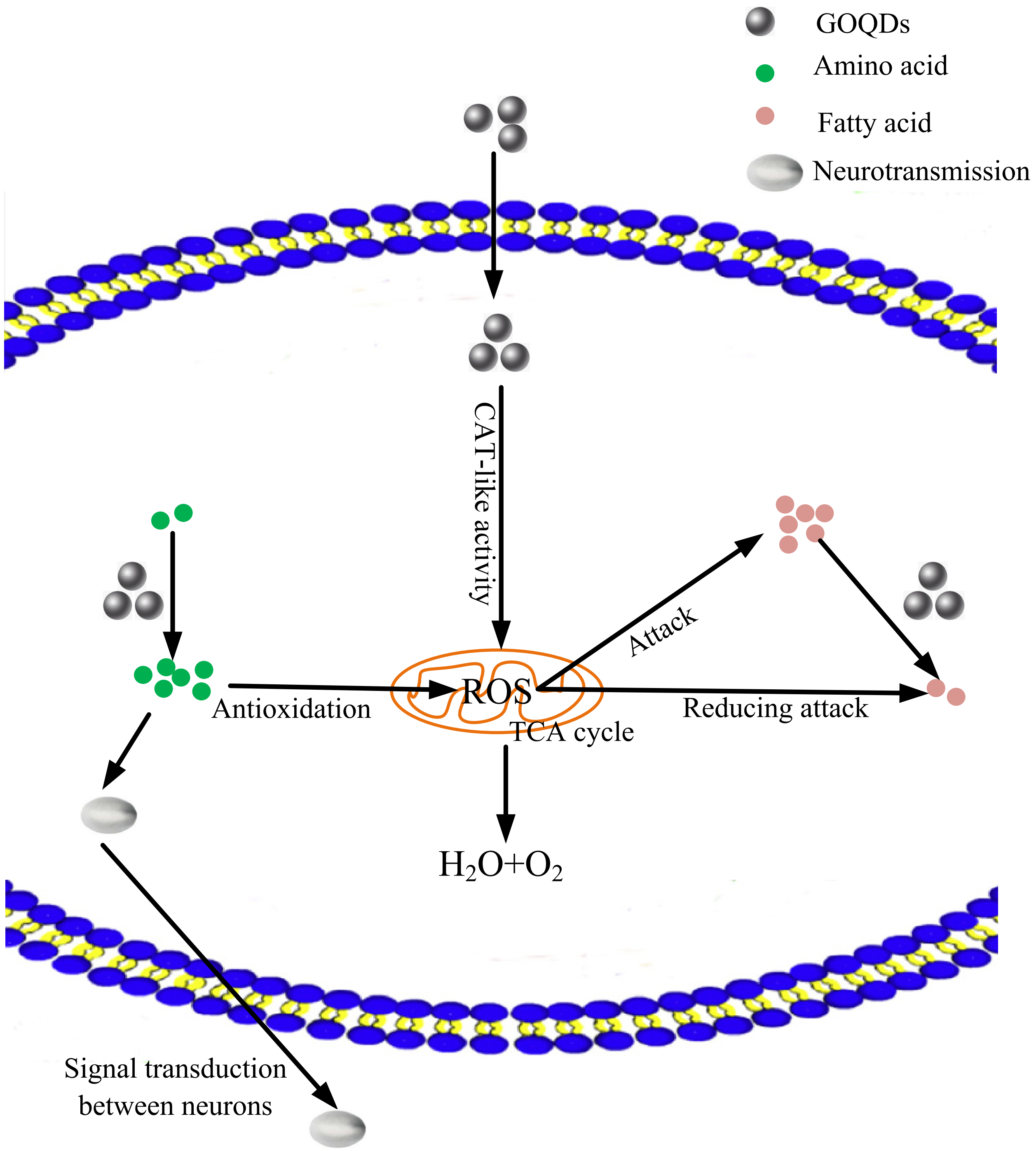

Figure S10 Diagram of GOQDs protecting PC12 cells and larval zebrafish from neurotoxicity and the underlying mechanism. Two green balls to six represents the upregulation of amino acids. In

contrast, six pink balls to two represents the downregulation of fatty acids. CAT, catalase.

References

[1] C. C. Ge, G. Fang, X. M. Shen, Y. Chong, W. G. Wamer, X. F. Gao, Z. F. Chai, C. Y. Chen, J. J. Yin, *ACS Nano* **2016**, *10*, 10436.

[2] D. A. Jasim, S. Murphy, L. Newman, A. Mironov, E. Prestat, J. McCaffrey, C. Ménard-Moyon, A. F. Rodrigues, A. Bianco, S. Haigh, R. Lennon, K. Kostarelos, *ACS Nano* **2016**, *10*, 10753.

[3] B. C. Qiu, Y. X. Deng, M. M. Du, M. Y. Xing, J. L. Zhang, *Sci. Rep.* **2016**, *6*, 10.
